# An Integrated Management System for Noncommunicable Diseases Program Implementation in a Sub-Saharan Setting

**DOI:** 10.3390/ijerph182111619

**Published:** 2021-11-04

**Authors:** Maria Agata Miselli, Francesco Cavallin, Samwel Marwa, Bruno Ndunguru, Rehema John Itambu, Katunzi Mutalemwa, Monica Rizzi, Giulia Ciccarelli, Simone Conte, Stefano Taddei, Gaetano Azzimonti, Giovanni Putoto, Giovanni Fernando Torelli

**Affiliations:** 1 Doctors with Africa CUAMM, Tosamaganga, Iringa P.O. Box 11, Tanzania; a.miselli@cuamm.org (M.A.M.); r.itambu@cuamm.org (R.J.I.); mutalemwa6@gmail.com (K.M.); monica.rizzi2@aovr.veneto.it (M.R.); cuamm@cuamm.org (G.C.); simone.conte@aovr.veneto.it (S.C.); g.azzimonti@cuamm.org (G.A.); 2 Department of Medicine, Tosamaganga District Designated Hospital, Tosamaganga, Iringa P.O. Box 11, Tanzania; 3 Independent Statistician, 36020 Solagna, Italy; cescocava@libero.it; 4 District Medical Office, Iringa District Council, Iringa P.O. Box 162, Tanzania; ded@iringadc.go.tz (S.M.); brnndunguru@gmail.com (B.N.); 5 Department of Internal Medicine, University of Pisa, 56122 Pisa, Italy; stefano.taddei@med.unipi.it; 6 Doctors with Africa CUAMM, 35121 Padua, Italy; g.putoto@cuamm.org; 7 Doctors with Africa CUAMM, Dar es Salaam P.O. Box 23447, Tanzania; 8 Department of Hematology, Oncology and Dermatology, Policlinico Umberto 1, 00161 Rome, Italy

**Keywords:** Sub-Saharan Africa, noncommunicable diseases, hypertension, diabetes

## Abstract

Morbidity and mortality due to noncommunicable diseases (NCDs) are growing exponentially across Tanzania. The limited availability of dedicated services and the disparity between rural and urban areas represent key factors for the increased burden of NCDs in the country. From March 2019, an integrated management system was started in the Iringa District Council. The system implements an integrated management of hypertension and diabetes between the hospital and the peripheral health centers and introduces the use of paper-based treatment cards. The aim of the study was to present the results of the first 6 months’ roll-out of the system, which included 542 patients. Data showed that 46.1% of patients returned for the reassessment visit (±1 month), more than 98.4% of patients had blood pressure measured and were checked for complication, more than 88.6% of patients had blood sugar tested during follow-up visit, and blood pressure was at target in 42.8% of patients with hypertension and blood sugar in 37.3% of diabetic patients. Most patients who were lost to follow-up or did not reach the targets were those without medical insurance or living in remote peripheries. Our findings suggest that integrated management systems connecting primary health facilities and referral hospitals may be useful in care and follow-up of patients with hypertension and diabetes.

## 1. Introduction

Noncommunicable diseases (NCDs) represent a group of chronic conditions, including cardiovascular diseases, cancer, chronic respiratory diseases, and diabetes, which account for 71% of all deaths worldwide, equivalent to 41 million people each year [1,2]. A large proportion of these deaths occur in low- and middle-income countries (LMICs), where about 700 million people still experience extreme levels of poverty [2,3]. The link between illness and poverty is well documented, as it is the role played by NCDs and injuries (NCDIs) in the suffering and death of the poorest populations [4,5,6].

In Tanzania, the burden of NCDIs has doubled in the past 25 years and accounts for 41% of all disability-adjusted life years (DALYs) [7]. While 80% of the global NCDIs burden is generally associated with lifestyle components (smoking habits, alcohol abuse, sedentary lifestyle, poor diet), the majority of NCDIs DALYs occurring in Tanzania cannot be explained by behavioral or metabolic risk factors [7,8]. The lack of treatment of conditions leading to chronic diseases, the linkage between infectious diseases and NCDs, and the limited availability of NCDIs services (which are mainly concentrated in hospitals and urban settings) may play crucial roles in this setting [9,10,11,12]. The Tanzania NCDI Poverty Commission reached the conclusion that the response to NCDIs among the poorest should consider socioeconomic indices, address material poverty, integrate models of health service delivery strategy that connect rural and urban areas, and complement the existing agenda focused on the prevention of emerging behavioral risk factors [13,14,15,16].

Similar to other sub-Saharan countries, hypertension is the most common NCD in Tanzania and impacts approximately 25% of the adult population [7,17,18,19,20], representing the leading cause of death after HIV and the leading cause of death due to NCDs [7,21]. Diagnosed individuals often are neither in blood pressure treatment nor seeking for care, and people living in rural areas are more likely to be unaware of their hypertension and therefore are less likely to be on treatment [7]. A major role is also played by diabetes, with a prevalence of 9% among adults 25–64 years old [7,15], and very high incidence of complications [22,23]. The STEPs survey of the World Health Organization (WHO) performed in 2012 revealed that three-quarters of participants with hypertension or diabetes were never previously diagnosed, and less than half of those with a previous diagnosis were receiving treatment [7,17]. Of note, individuals in the lower wealth quintile, those less educated, and those from rural areas were less likely to have prior blood glucose or blood pressure tested. Moreover, it is well known that treatment adherence and clinical follow-up play a crucial role in the management of NCDs, but health systems in many sub-Saharan countries have limited capacity of long-term continuous management of such patients [7].

Since 2016, Doctors with Africa CUAMM [24], in partnership with local authorities, has been running a dedicated clinical program at Tosamaganga District Designated Hospital (DDH), Iringa District Council (DC), Iringa Region. As the health system could not regularly engage patients for follow-up, there was the need to implement a new management system based on the systematic link between the hospital and the peripheral health units of the district. The experience conducted in Tanzania in HIV care [25], as well as old [26] and newly [27] released WHO packages for NCDs care implementation, represents key models of inspiration.

In March 2019, an integrated management system of hypertension and diabetes has started in collaboration with the local authorities of Iringa DC and Tosamaganga DDH. The purpose of this study was to present the results of the first 6 months’ roll-out of the system.

## 2. Materials and Methods

### 2.1. Study Design

This prospective cohort study presents the results of the first 6 months’ roll-out of an integrated management system for patients with hypertension and/or diabetes in Iringa DC (Tanzania). All patients who were enrolled in the new model between March and September 2019 were included in the study. Data on follow-up were retrieved in June 2020. The study was approved by the National Institute for Medical Research (NIMR/HQ/R.8a/Vol. IX/3294). The study was conducted in accordance with the principles of the Declaration of Helsinki, and all participants gave their written informed consent to have their anonymized data used for scientific purpose.

### 2.2. Setting

According to the World Health Organization (WHO), diabetes and hypertension affect a large proportion of Tanzanian adults; adult population includes 14% of tobacco users, 9% alcohol abusers, and 7% obese [18].

Iringa DC is located in a rural area 500 km southwest of Dar es Salaam, has a population of about 358,000 inhabitants, distributed in a surface area of 20,414 km^2^, and the health care system includes a District Hospital (Tosamaganga DDH), 10 health centers (HCs), and 67 dispensaries. The Tanzanian health system has a hierarchical and decentralized structure [28]. Each district has a designated hospital (primary level) which is the referral hospital for health centers and dispensaries within the district. District hospitals refer to a regional hospital (secondary level), and all regional hospitals to zonal and national hospitals (tertiary level). Administrative data include 336 hospitals, 907 health centers, and 7247 dispensaries, distributed in 26 regions for a population of more than 56 million inhabitants. Since October 2016, Doctors with Africa CUAMM and the Iringa DC have set up an outpatient service exclusively dedicated to patients suffering from NCDs at the outpatient department (OPD) of Tosamaganga DDH. People attending the NCDs clinic arrive from all over Iringa DC, coming from 134 different villages, and are referred from all 10 HCs of the district.

### 2.3. Participants

Eligible subjects were adults (age ≥ 18 years) with hypertension and/or diabetes who were followed-up at Tosamaganga DDH and in all 10 district HCs. Patients with both new and known diagnosis of hypertension and/or diabetes were invited to attend the Tosamaganga DDH NCDs clinic for the registration visit. Patients with all types of diabetes were included in the study. Pregnant women were excluded.

### 2.4. The Integrated Management System

**Figure 1:**
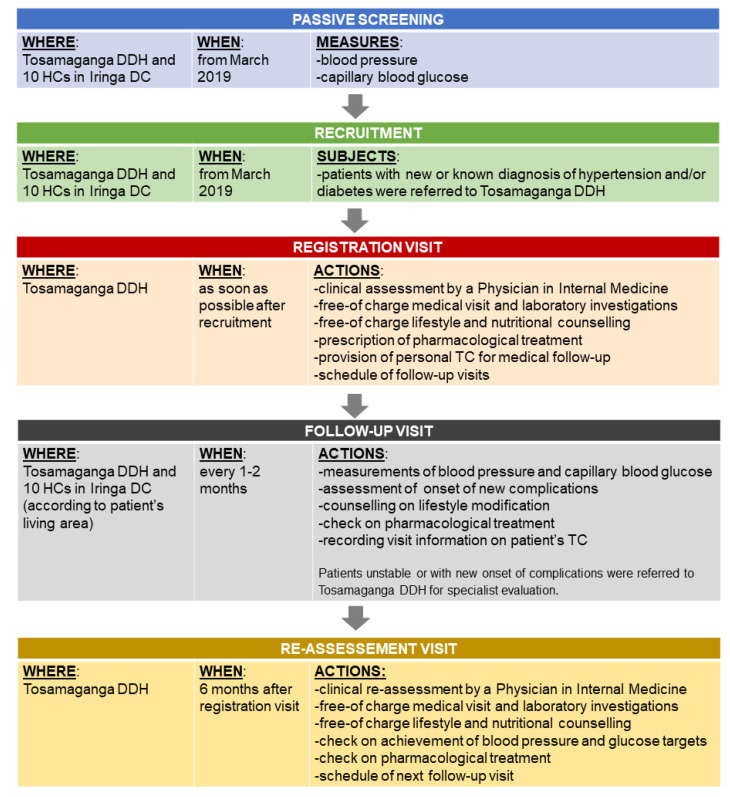
Scheme of the integrated management system (full description in Supplementary Table S1).

The implementation of an integrated management system of hypertension and diabetes (Figure 1) was started at the beginning of March 2019, after the finalization of the Protocol of Cooperation Agreement among Iringa DC, Tosamaganga DDH, and Doctors with Africa CUAMM. The Protocol of Cooperation Agreement was conceived to reinforce and improve the health system of Iringa DC; in particular, regarding the prevention and treatment of NCDs at hospital and HC levels, with the purpose of warranting access, quality, and equitable health care for the population of the district.

Before the implementation, blood pressure and blood sugar were measured randomly (according to patient’s request or health care staff decision, equipment availability, and patient’s willingness to cover the costs) and follow-up was not systematically offered. In addition, patients were referred from HCs to the hospital, without any back-referral or information feedback to HCs.

**Figure 2:**
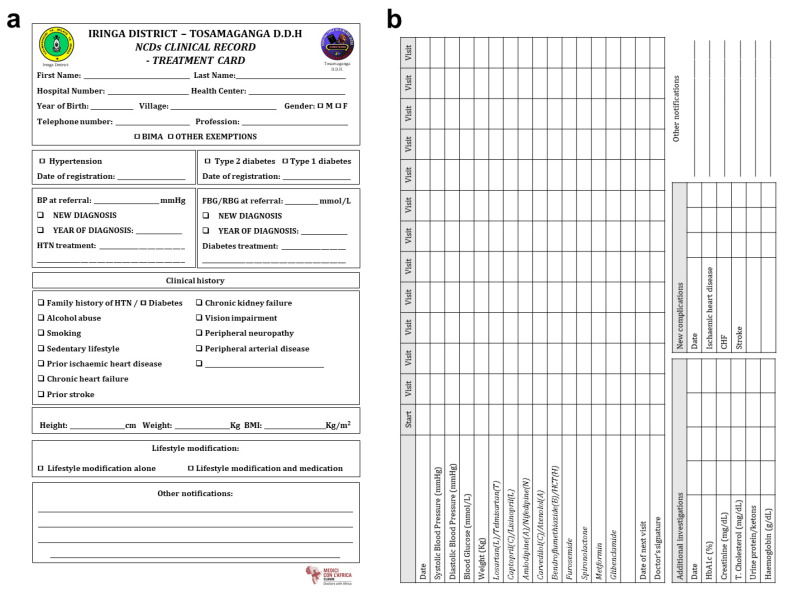
Template of the paper-based treatment cards. (**a**) Front of treatment card. (**b**) Back of treatment card.

The integrated system included the creation of pathways for patients and the implementation of the use of paper-based treatment cards (TCs) (Figure 2). Each patient (with either a new diagnosis or a previous diagnosis) underwent the initial assessment at Tosamaganga DDH, and all enrolled patients were supplied with personal TCs. Monthly follow-up visits were conducted at the hospital or the HCs, where clinical records and treatment information were regularly recorded in the TCs. The patient returned to the hospital for a reassessment visit every six months (±1 month). The reassessment visit was set at 6 months after registration because such a time span would have provided useful feedback on the roll-out of the integrated system with a reasonable frequency for the patient (monthly visit at the closest health center and travel to the referral hospital only twice a year). This cut-off time was inspired by the WHO dedicated package [27]. The implementation is fully described in Supplementary Table S1.

Screening and diagnosis of hypertension and diabetes were conducted according to national guidelines [29]. Lifestyle counselling and pharmacological treatment, as well as criteria for referral to higher level of care during follow-up, were provided according to national NCDs guidelines [30]. TC and treatment targets were directly inspired by WHO HEARTS technical package [27].

Patients’ registration and enrolment started on 18 March 2019, and the system is currently ongoing.

### 2.5. Outcome Measures

The outcome measures included (i) adherence to reassessment visit (±1 month) at Tosamaganga DDH, (ii) patient attendance and quality of data collection during follow-up visits, (iii) achievement of treatment target at reassessment visit (±1 month), and (iv) occurrence of complications (stroke, diabetic foot, vision impairment, heart failure, and heart ischemia) during follow-up.

### 2.6. Data Collection

The records of patients who were enrolled between 18 March 2019 and 18 September 2019 were used for this study. Data were retrieved from medical records noted on patients’ TCs and entered in an anonymized database for the analysis. The health care staff on duty was responsible for data collection on patients’ TC, which was checked by the medical doctor before data entry in the study database. Available data included demographics, and information from registration visit, follow-up visits, and reassessment visit. Data on follow-up were retrieved on 18 June 2020, to ensure an adequate follow-up for patients included in the study.

### 2.7. Statistical Analysis

Data were summarized as median and interquartile range (continuous data) or frequency and percentage (categorical data). Categorical data were compared between groups using chi square test or Fisher’s exact test, while continuous data were compared using Mann–Whitney test or Kruskal–Wallis test. Correlation between continuous data was assessed using Spearman rank correlation coefficient. The change (from baseline to the six-month reassessment visit (±1 month)) in the proportion of hypertensive patients with target blood pressure and of diabetic patients with target fasting blood glucose was evaluated using McNemar test. All tests were two-sided, and a *p*-value less than 0.05 was considered statistically significant. Statistical analysis was performed using R 4.0 (R Foundation for Statistical Computing, Vienna, Austria) [31].

## 3. Results

### 3.1. Patients

The study included 542 patients (134 males and 408 females; median age 61 years) who were enrolled between March and September 2019. Patient characteristics are shown in Table 1.

**Figure 3:**
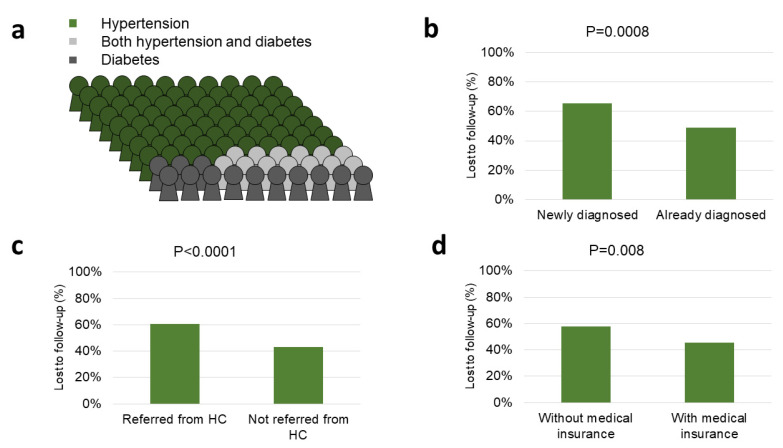
Diagnosis in 542 patients who were enrolled between March and September 2019 (**a**); lost to follow-up was more frequent in newly diagnosed patients (**b**); lost to follow-up was more frequent in patients referred from district health centers (**c**); lost to follow-up was more frequent in patients without medical insurance (**d**).

Hypertension was found in 475 patients (Figure 3A): 132 of them (27.8%) were new diagnoses, 304 (64.0%) were already in treatment for hypertension, and 39 (8.2%) had already a diagnosis of hypertension but were not receiving any treatment. Blood pressure (BP) was within the target range (systolic BP < 140 mmHg and diastolic BP < 90 mmHg) in 88/335 patients with previous hypertension diagnosis (26.3%).

Diabetes was found in 139 patients (Figure 3A): 33 of them (23.7%) were new diagnoses, 94 (67.6%) were already in treatment for diabetes, and 12 (8.6%) had already a diagnosis of diabetes but were not receiving any treatment. Fasting blood glucose was (<7 mmol/L) in 30/106 patient with previous diabetes diagnosis (28.3%).

### 3.2. Adherence to Follow-Up

At the time of the analysis, 250 patients (46.1%) returned for follow-up visits, while three patients (0.6%) died, and 289 patients (53.3%) were lost to follow-up (i.e., never returned for follow-up visits). Loss to follow-up was 54.1% among hypertensive patients (218/403), 59.7% among diabetic patients (40/67), and 43.1% among patients with hypertension and diabetes (31/82). Median number of visits was five (IQR 4–6) in patients who returned for reassessment visit (±1 month) at Tosamaganga DDH after six months.

Loss to follow-up was more frequent in newly diagnosed patients (95/145, 65.5% vs. 194/397, 48.9%, *p* = 0.0008; Figure 3B), patients referred from health centers (189/312, 60.6% vs. 99/229, 43.2%, *p* < 0.0001; Figure 3C), and in those without medical insurance (202/351, 57.5% vs. 86/190, 45.3%, *p* = 0.008; Figure 3D). Of note, new diagnoses were more frequent among patients referred from health centers (107/312, 34.3% vs. 37/229, 16.1%; *p* < 0.0001). Loss to follow-up visits was not associated with diagnosis (*p* = 0.12), age (*p* = 0.92), sex (*p* = 0.11), family history of hypertension (*p* = 0.69), or family history of diabetes (*p* = 0.84) (Supplementary Table S2).

In patients who returned for follow-up reassessment visit (±1 month) at Tosamaganga DDH, the number of visits was not associated with diagnosis (*p* = 0.41), being referred from health centers (*p* = 0.39), medical insurance (*p* = 0.83), age (*p* = 0.80), sex (*p* = 0.29), family history of hypertension (*p* = 0.60), or family history of diabetes (*p* = 0.65) (Supplementary Table S3).

### 3.3. Data Collection during Follow-Up Visits

**Figure 4:**
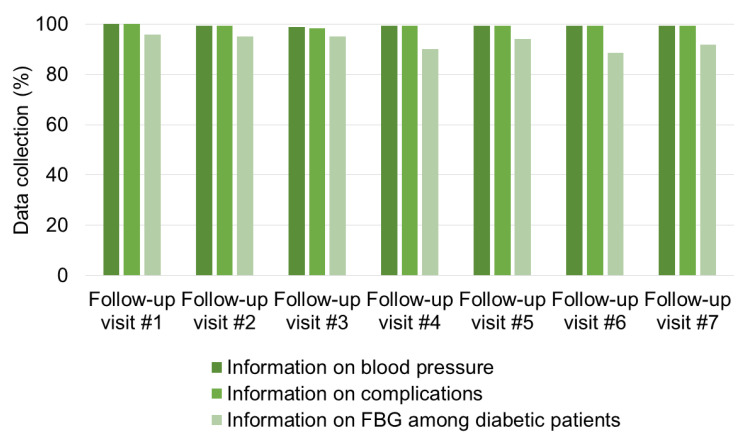
Data collection during follow-up visits.

During follow-up visits #1 to #7, patient attendance ranged between 149 and 188 patients (Supplementary Table S4). Almost all patients had their BP measured (98.9–100%) and were checked for complications (98.4–100%), while FBG was measured in 88.6–95.8% of diabetic patients (Figure 4).

## 4. Achievement of Treatment Target after Six Months of Follow-Up

**Figure 5:**
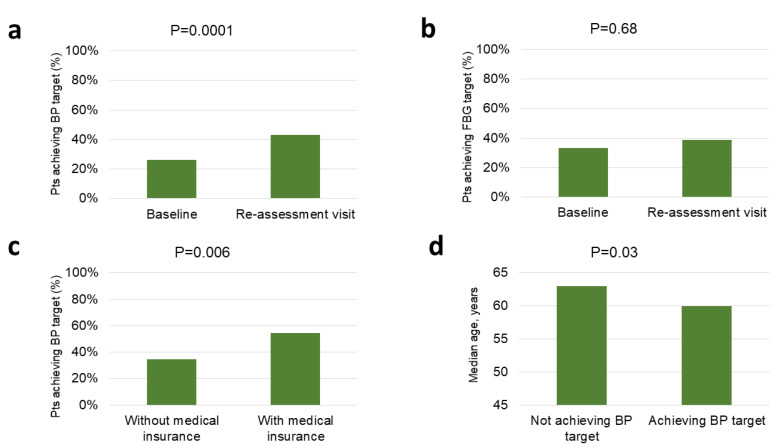
Patients with hypertension achieving blood pressure target at baseline and reassessment visit (±1 month) (**a**); patients with diabetes achieving fasting blood glucose target at baseline and reassessment visit (±1 month) (**b**); association between achieving blood pressure target at reassessment visit (±1 month) and medical insurance (**c**); association between achieving blood pressure target at reassessment visit (±1 month) and age (**d**).

Reassessment visit (±1 month) at Tosamaganga DDH was attended by 231 patients (42.6%). Target BP (systolic BP < 140 mmHg and diastolic BP < 90 mmHg) was achieved in 89/208 hypertensive patients (42.8%), with an increase of the proportion of those achieving target BP from 53/202 (25.5%) at baseline to 87/202 (41.8%) at reassessment visit (±1 month) (*p* = 0.0001) (Figure 5A). Achieving target BP was more frequent in patients with medical insurance (46/84, 54.8% vs. 43/124, 34.7%, *p* = 0.006; Figure 5C) or younger age (median 60 vs. 63 years, *p* = 0.03; Figure 5D), while it was not associated with new diagnosis (*p* = 0.61), being referred from health centers (*p* = 0.07), sex (*p* = 0.95), or family history of hypertension (*p* = 0.15) (Supplementary Table S5).

Target FBG (FBG < 7 mmol/L) was achieved in 22/59 diabetic patients (37.3%), without statistically significant change in the proportion of those target FBG from baseline (19/57, 33.3%) to reassessment visit (22/57, 38.6%) (*p* = 0.68) (Figure 5B). Achieving target FBG was not associated with new diagnosis (*p* = 0.51), being referred from health centers (*p* = 0.32), medical insurance (*p* = 0.76), age (*p* = 0.17), sex (*p* = 0.35), or family history of diabetes (*p* = 0.99) (Supplementary Table S6).

### Complications during Follow-Up

During the first six months of follow-up, stroke occurred in two patients, diabetic foot in four patients, vision impairment in two patients, heart failure in five patients, and heart ischemia in none. Further description of these patients is reported in Supplementary Table S7.

## 5. Discussion

The current study aimed to present the results of the first 6 months’ roll-out of an integrated management system of hypertension and diabetes in Tanzania, based on the use of paper-based treatment cards belonging to the patient, the tight connection between primary health facilities and referral hospitals, and the ownership of the program to local authorities (district/regional medical officers).

The study confirmed that in Tanzania, many patients affected by hypertension and diabetes do not receive any treatment and only about one fourth of those actually in treatment reach the target [17,32]. During the first six months of enrolment, approximately five new cases of hypertension and one new case of diabetes were registered each week. This represents a consistent number of chronic patients to deal with for the type of health services actually present in rural areas of Tanzania.

Participant characteristics were typical of rural areas, with 57.6% peasants and the rest mainly retired. The old age of the study group is not surprising as chronic diseases are common in adults, although it appears quite high, taking into consideration that life expectancy in Tanzania is 64 years [33]. Finally, we believe that the higher female presence in the program could probably reflect the higher attendance of health facilities by the female population.

A large group of patients enrolled in the study were lost to follow-up (53.3%); the majority of them were those without medical insurance or who were referred from health centers, suggesting that poverty and distance were the most relevant contributing factors preventing patients to return to the hospital. Of note, loss to follow-up was also more common in newly diagnosed patients, which were more frequently referred from health centers. While no data are available in the literature concerning attendance and follow-up visit for NCDs in Tanzania, we believe that this issue is probably one of the most relevant in the management and care of chronic diseases. In light of the specific setting of our intervention, it is noteworthy that almost half of the patients returned for the reassessment visit (±1 month) during follow-up; additionally, patients not returning to the District Hospital for the reassessment visit ±1 month may be still receiving the necessary care and treatment at their health center of origin, confirming the importance of decentralizing the health program in the context of chronic care in order to reduce distance and improve accessibility to health care.

Health insurance coverage is still low in Tanzania; as of 2019, only 32% of Tanzanians had health insurance coverage, of which 8% have subscribed to National Health Insurance Fund (NHIF), 23% are members of Community Health Fund, and 1% are members of private health insurance companies [34]. Low insurance coverage leads to overreliance on direct payment, which is among the fundamental problems that restrain the move towards universal health coverage in many developing countries [35]. Direct payment leads to high levels of inequity, in most cases denying the poorest to access the needed health care [36]. The NHIF was established in 1999 and a steady increase in coverage, from 2% of the total population in 2001 to 8% in 2019, has been observed [34]; nevertheless, the study results underline the need to continue reforming the health care system and improve health insurance coverage with the intention of increasing universal access to health services to the poor and those living in marginalized rural areas.

For those patients that had successfully performed reassessment (±1 month), general satisfactory implementation of the system was observed, especially concerning attendance to follow-up visits and correct documentation on treatment cards. In fact, the mean number of visits per patient in the 6-month period was five, and almost all patients had their blood pressure measured (98.9–100%) during follow-up visits, while fasting blood glucose was measured in 88.6–95.8% of diabetic patients. This difference was probably due to glucose test strips availability. The high percentage of patients who had blood pressure and fasting blood sugar measured and were checked for complications during follow-up visit (including those performed at health centers), indicates that the availability of simple instruments, the tight connection between central hospital and peripheral health facilities, and the provision of adequate training can improve the management of NCDs in Tanzania.

At last, when evaluating the clinical outcomes through the achievement of guidelines targets for hypertension and diabetes, it became evident that only a minority of patients enrolled in the study succeeded to achieve the targets after six months of follow-up. This is a well-known challenge which is already documented in other studies; in the 2012 Tanzania STEPS Survey, for example, only 42.4% of patients treated for hypertension had systolic blood pressure <140 mmHg and diastolic blood pressure <90 mmHg [15], while in other studies, the percentage of patients at target was significantly lower [32]. When exploring the potential contributors to the achievement of these goals, once again it was shown that health insurance holders, together with younger patients, were more likely to satisfy clinical targets for hypertension. Moreover, the provenance from health centers seemed to play a negative role on clinical target achievement, probably due to drugs availability at the peripheral level that was still uncertain and limited to few drugs categories. From baseline to reassessment visit (±1 month), the proportion of hypertensive patients who achieved target blood pressure increased significantly, while a small nonsignificant increment was observed in the proportion of diabetic patients who achieved target fasting blood glucose. Nevertheless, the management of patients with diabetes is still challenging due to the great economic burden and the need for an acceptable level of education to self-manage insulin therapy.

Finally, we observed very low prevalence of complications during follow-up, though the short period of observation suggests caution and a need for long-term assessment. However, the integrated system favored the connection between the hospital and the health centers, and patients with new onset of complications were promptly referred to Tosamaganga DDH for specialist evaluation.

This study has some limitations that should be considered by the reader. First, the limited duration of the follow-up in the study (6 months) suggests caution in the interpretation of the adherence to follow-up and the occurrence of complications. However, the six-monthly control was suggested by the WHO HEARTS technical package (hypertensive subjects) [27] and the Tanzanian Desk Guide (diabetic subjects) [30]. In addition, information on the reasons for loss to follow-up was not available but would have been useful to plan adequate actions for improving follow-up adherence. Second, the generalizability of the findings should be restricted to similar settings. Third, the study would have benefited from the comparison of pre- and post-implementation periods to emphasize the importance of the change. Unfortunately, previous data were not available because systematic data collection was not performed before program implementation. Future developments of the integrated system will include the contacting of patients skipping follow-up visits (to remind them of scheduled visits, to understand the reasons for unattendance, and to plan adequate actions for improving the follow-up adherence) and the systematic check on medication adherence and pharmacovigilance during follow-up visits. Of note, the implementation of an electronic database system linking the referral hospital and the health centers would notably improve the management of NCD patients living in remote areas. In addition, an update of the study over a longer time span is warranted to provide more reliable data on follow-up and complications, and to assess the effect of further developments.

## 6. Conclusions

These results confirm that gaps in the control of noncommunicable diseases are still large in Tanzania. Nevertheless, the analysis performed on this integrated management system suggests that health system interventions are possible and should be properly designed, taking into consideration socioeconomic indicators and proposed models of health delivery strategy owned by local authorities tightly connecting primary health facilities and referral hospitals. Should these positive results be confirmed after long-term assessment, similar programs might be taken into consideration for implementation on a larger scale in Tanzania.

## Figures and Tables

**Table 1:** Characteristics of 542 patients who were enrolled between March and September 2019.

Variable	All Patients	Hypertensive Patients	Diabetic Patients	Hypertensive and Diabetic Patients
No. of subjects	542	403	67	72
Age, years ^a,b^	61 (53–69)	62 (55–70)	52 (44–60)	61 (54–65)
Males:females	134:408	92:311	21:46	21:51
Personal insurance holders ^b^	190 (35.1)	129 (32.1)	27 (40.3)	34 (47.2)
Referred from district health centers ^b^	312 (57.7)	249 (61.9)	37 (55.2)	26 (36.1)
Job:				
Peasant	312 (57.6)	253 (62.8)	29 (43.3)	30 (41.7)
Employed	70 (12.9)	39 (9.7)	15 (22.4)	16 (22.2)
Unemployed ^c^	16 (3.0)	7 (1.7)	6 (9.0)	3 (4.2)
Retired	100 (18.4)	77 (19.1)	9 (13.4)	14 (19.4)
Other/no response	44 (8.1)	27 (6.7)	8 (11.9)	9 (12.5)
Family history of hypertension ^b^	159 (29.4)	125 (31.0)	15 (22.7)	19 (26.4)
Family history of diabetes	67 (12.4)	30 (7.4)	17 (25.4)	20 (27.8)
Regular daily alcohol consumption	177 (32.7)	144 (35.7)	15 (22.4)	18 (25.0)
Regular daily smoking habits	26 (4.8)	22 (5.4)	2 (3.0)	2 (2.8)
Sedentary lifestyle (>5 h spent seated daily)	140 (25.8)	100 (24.8)	20 (29.9)	20 (27.8)
Prior heart attack	7 (1.3)	7 (1.7)	0 (0.0)	0 (0.0)
Chronic heart failure	59 (10.9)	53 (13.1)	0 (0.0)	6 (8.3)
Prior stroke	38 (7.0)	28 (6.9)	2 (3.0)	8 (11.1)
Vision impairment	25 (4.6)	12 (3.0)	7 (10.4)	6 (8.3)
Diabetic foot	0 (0.0)	0 (0.0)	0 (0.0)	0 (0.0)

Data expressed as no. (%) or ^a^ median (IQR). Data not available in ^b^ 1 patient. ^c^ Including students and housewives.

## Data Availability

All data are fully available without restriction. All relevant data are within the manuscript and its Supporting Information files.

## Supplementary Materials

The following are available online at https://www.mdpi.com/article/10.3390/ijerph182111619/s1, Table S1: Scheme of model implementation, Table S2: Factors associated with lost to follow-up (i.e., not returning for re-assessment visit at Tosamaganga DDH), Table S3: Factors associated with number of visits among patients who returned for follow-up, Table S4: Data collection during follow-up visits, Table S5: Factors associated with achieving target blood pressure in hypertensive patients, Table S6: Factors associated with achieving target Fasting Blood Glucose in diabetic patients, Table S7: Characteristics of patients who experience a complication during the first six-months of follow-up.
