# Supplementary material for: An Integrated Management System for Noncommunicable Diseases Program Implementation in a Sub-Saharan Setting
Source: Int J Environ Res Public Health. 2021 Nov 4;18(21):11619. doi: 10.3390/ijerph182111619 (PMC8583607; doi:10.3390/ijerph182111619)
Supplement: Supplementary file 1 [file ijerph-18-11619-s001.zip › Supplementary Table S1.pdf]

Supplementary Table S1. Scheme of model implementation

Step	Where	When	Who	Description
Passive screening	Tosamaganga DDH and 10 HCs in Iringa DC	From March 2019	Eligible subjects were identified using passive case finding approach of subjects attending the Hospital and the district HCs. The screening was supported with periodical supervisions by study personnel (every three months).	Blood pressure was measured using a manual sphygmomanometer after 5 minutes of rest, in seated position with patient's arm elevated at the level of the heart. High values were confirmed by double checking. Presence of hypertension was defined as blood pressure ≥ 140/90 mmHg according to national and international guidelines (23-25).<br>Capillary blood glucose (Glucoplus Inc, Canada) was performed on site by finger prick by a medical officer. Presence of diabetes was defined as Fasting Blood Glucose (FBG) ≥ 126 mg/dl or Random Blood Glucose (RBG) ≥ 200 mg/dl according to national and international guidelines (24, 26).
Recruitment	Tosamaganga DDH and 10 HCs in Iringa DC	From March 2019	All patients with new or known diagnosis of hypertension and/or diabetes were considered eligible and referred to Tosamaganga DDH for registration	Patients were referred to Tosamaganga DDH for the clinical assessment and the registration visit.
Registration visit	Tosamaganga DDH	As soon as possible after recruitment	Recruited patients that successfully reached Tosamaganga DDH for registration visit and were asked to participate in the study.	At Tosamaganga DDH, a Physician in Internal Medicine was responsible for the clinical assessment (where hypertension was checked for confirmation) and the registration visit. After signing the informed consent form in Swahili language, demographics, clinical history, and hypertension/diabetes treatment before registration were collected.<br>All patients underwent medical visit (for screening of acute and chronic complications) and laboratory investigations (including FBG, haemachrome and white blood cell count, total cholesterol, creatinine, urine stick for ketones and proteins).<br>A nutritionist provided lifestyle counselling and nutritional advice. During a head-to-head interview, the nutritionist investigated the lifestyle/nutritional habits of each patient, and provided personalized suggestions and indications (with also aid of written material) to improve patient's health and habits, but also his/her understanding of the disease.<br>The medical visit and the laboratory investigations were free-of charge.<br>Pharmacological treatment was prescribed as needed and according to NCDs national guidelines (24). Treatment was started accordingly to the total cardiovascular risk approach and always through patient's engagement in the treatment process.<br>Patients were finally provided with personal TC for medical follow-up at the hospital/HC level and a follow-up visit was scheduled.

Follow-up visit	Tosamaganga DDH and 10 HCs in Iringa DC (according to patient's living area)	Every 1-2 months	Enrolled patients	The staff recorded visit information (date of visit, systolic blood pressure, diastolic blood pressure, FBG in diabetic patients, new complications, treatment, and date of next follow-up visit) on patient's TC.<br>Patients were also counselled on lifestyle modification, while those unstable or with new onset of complications were referred to Tosamaganga DDH for specialist evaluation.<br>Pharmacological treatment was prescribed for the entire period between two consecutive visits.<br>There were no dedicated resources for the visits, that were performed by the current staff as per availability.<br>Supervision was conducted every three months in all district HCs to monitor the correct use of TCs and potential issues during patient's follow-up.
Re-assessment visit	Tosamaganga DDH	6 months after the registration visit	Enrolled patients	Six months (± 1 month) after the registration visit, patients were referred to Tosamaganga DDH where the Physician in Internal Medicine was responsible for the clinical re-assessment.<br>Patients underwent medical visit (for screening of acute and chronic complications) and laboratory investigations (including FBG, haemachrome and white blood cell count, total cholesterol, creatinine, urine stick for ketones and proteins). A nutritionist provided lifestyle counselling and nutritional advice.<br>The patient was informed whether he/she has reached blood pressure and/or FBG treatment target. BP target was systolic BP <140 mmHg and diastolic BP <90 mmHg, FBG target was <126 mg/dl (24-26).<br>The medical visit and the laboratory investigations were free-of charge.
