# Supplementary material for: An Integrated Management System for Noncommunicable Diseases Program Implementation in a Sub-Saharan Setting
Source: Int J Environ Res Public Health. 2021 Nov 4;18(21):11619. doi: 10.3390/ijerph182111619 (PMC8583607; doi:10.3390/ijerph182111619)
Supplement: Supplementary file 1 [file ijerph-18-11619-s001.zip › Supplementary Table S2 rev1.pdf]

Supplementary Table S2. Factors associated with lost to follow-up (i.e. not returning for re-assessment visit at Tosamaganga DDH)

	Patients not lost to follow-up	Patients lost to follow-up	p-value
No. of subjects	253	289	-
Age, years [ab]	61 (53-69)	61 (53-68)	0.92
Males	54 (21.3)	80 (27.7)	0.11
Personal insurance holders [b]	104 (41.1)	86 (29.9)	0.008
Referred from district health centers [b]	123 (48.6)	189 (65.6)	<0.0001
Diagnosis:<br>Hypertension<br>Diabetes<br>Both hypertension and diabetes	 185 (73.1)<br>27 (10.7)<br>41 (16.2)	 218 (75.4)<br>40 (13.8)<br>31 (10.7)	0.12
Already diagnosed<br>Newly diagnosed	203 (80.2)<br>50 (19.8)	194 (67.1)<br>95 (32.9)	0.0008
Family history of hypertension [b]	77 (30.4)	82 (28.5)	0.69
Family history of diabetes	30 (11.9)	37 (12.8)	0.84

Data expressed as No. (%) or [a] median (IQR). Data not available in [b]1 patient.
