# Supplementary material for: An Integrated Management System for Noncommunicable Diseases Program Implementation in a Sub-Saharan Setting
Source: Int J Environ Res Public Health. 2021 Nov 4;18(21):11619. doi: 10.3390/ijerph182111619 (PMC8583607; doi:10.3390/ijerph182111619)
Supplement: Supplementary file 1 [file ijerph-18-11619-s001.zip › Supplementary Table S3 Rev1.pdf]

Supplementary Table S3. Factors associated with number of visits among patients who returned for follow-up

	Number of visits	p-value
Age, years [a]	0.02	0.80
Females<br>Males	6 (4-6)<br>5 (4-7)	0.29
Personal insurance:<br>Non-holders<br>Holders	 5 (4-6)<br>6 (4-6)	0.83
Referred from health centers:<br>No<br>Yes	 6 (5-6)<br>5 (4-6)	0.39
Diagnosis:<br>Hypertension<br>Diabetes<br>Both hypertension and diabetes	 6 (4-7)<br>5 (4-6)<br>5 (4-6)	0.41
Family history of hypertension:<br>No<br>Yes	 6 (4-7)<br>5 (4-6)	0.60
Family history of diabetes:<br>No<br>Yes	 5 (4-6)<br>6 (4-7)	0.65

Data expressed as median (IQR) or [a] Spearman correlation coefficient.
