# Supplementary material for: An Integrated Management System for Noncommunicable Diseases Program Implementation in a Sub-Saharan Setting
Source: Int J Environ Res Public Health. 2021 Nov 4;18(21):11619. doi: 10.3390/ijerph182111619 (PMC8583607; doi:10.3390/ijerph182111619)
Supplement: Supplementary file 1 [file ijerph-18-11619-s001.zip › Supplementary Table S4.pdf]

Supplementary Table S4. Data collection during follow-up visits

Follow-up visit	Attending patients	Information on blood pressure in all patients	Information on complications in all patients	Information on FBG in diabetic patients
#1	180	180 (100.0%)	180 (100.0%)	45/48 (95.8%)
#2	170	169 (99.4%)	169 (99.4%)	40/42 (95.2%)
#3	188	186 (98.9%)	185 (98.4%)	39/41 (95.1%)
#4	164	163 (99.4%)	163 (99.4%)	36/40 (90.0%)
#5	179	178 (99.4%)	178 (99.4%)	48/51 (94.1%)
#6	160	159 (99.4%)	159 (99.4%)	39/44 (88.6%)
#7	149	148 (99.3%)	148 (99.3%)	34/37 (91.9%)

Complications included stroke, heart ischemia, diabetic foot, vision impairment and heart failure.
