# Supplementary material for: An Integrated Management System for Noncommunicable Diseases Program Implementation in a Sub-Saharan Setting
Source: Int J Environ Res Public Health. 2021 Nov 4;18(21):11619. doi: 10.3390/ijerph182111619 (PMC8583607; doi:10.3390/ijerph182111619)
Supplement: Supplementary file 1 [file ijerph-18-11619-s001.zip › Supplementary Table S5.pdf]

Supplementary Table S5. Factors associated with achieving target blood pressure in hypertensive patients

	Not achieving target blood pressure	Achieving target blood pressure	p-value
No. of subjects	119	89	-
Age, years [a]	63 (56-70)	60 (52-67)	0.03
Males	23 (19.3)	16 (18.0)	0.95
Personal insurance holders	38 (31.9)	46 (51.7)	0.006
Referred from district health centers	66 (55.5)	37 (41.6)	0.07
New diagnosis of hypertension	26 (21.8)	16 (18.0)	0.61
Family history of hypertension	33 (27.7)	34 (38.2)	0.15

Data expressed as No. (%) or [a] median (IQR).
