# Supplementary material for: An Integrated Management System for Noncommunicable Diseases Program Implementation in a Sub-Saharan Setting
Source: Int J Environ Res Public Health. 2021 Nov 4;18(21):11619. doi: 10.3390/ijerph182111619 (PMC8583607; doi:10.3390/ijerph182111619)
Supplement: Supplementary file 1 [file ijerph-18-11619-s001.zip › Supplementary Table S6.pdf]

Supplementary Table S6. Factors associated with achieving target Fasting Blood Glucose in diabetic patients

	Not achieving target Fasting Blood Glucose	Achieving target Fasting Blood Glucose	p-value
No. of subjects	37	22	-
Age, years [a]	60 (48-63)	62 (54-65)	0.17
Males	8 (21.6)	8 (36.4)	0.35
Personal insurance holders	14 (37.8)	10 (45.5)	0.76
Referred from district health centers	18 (48.7)	7 (31.8)	0.32
New diagnosis of diabetes	9 (24.3)	3 (13.6)	0.51
Family history of diabetes	8 (21.6)	5 (22.7)	0.99

Data expressed as No. (%) or [a] median (IQR).
