# Supplementary material for: An Integrated Management System for Noncommunicable Diseases Program Implementation in a Sub-Saharan Setting
Source: Int J Environ Res Public Health. 2021 Nov 4;18(21):11619. doi: 10.3390/ijerph182111619 (PMC8583607; doi:10.3390/ijerph182111619)
Supplement: Supplementary file 1 [file ijerph-18-11619-s001.zip › Supplementary Table S7 Rev1.pdf]

Supplementary Table S7. Characteristics of patients who experience a complication during the first six-months of follow-up

No. of subjects	13
Diagnosis:<br>Hypertension<br>Diabetes<br>Hypertension and diabetes	 6 (46.1)<br>4 (30.8)<br>3 (23.1)
Type of complications:<br>Heart failure<br>Diabetic foot<br>Stroke<br>Vision impairment	 5 (38.4)<br>4 (30.8)<br>2 (15.4)<br>2 (15.4)
Age, years [a]	64 (60-70)
Males: females	2:11
Personal insurance holders	4 (30.8)
Referred from district health centers	8 (61.5)
Job:<br>Peasant<br>Unemployed<br>Retired<br>Other/no response	 5 (38.4)<br>1 (7.7)<br>4 (30.8)<br>3 (23.1)
Family history of hypertension	2 (15.4)
Family history of diabetes	3 (23.1)
Regular daily alcohol consumption	2 (15.4)
Regular daily smoking habits	1 (7.7)
Sedentary lifestyle (>5 hours spent seated daily)	3 (23.1)

Data expressed as n (%) or [a] median (IQR).
